# Case report: one case of coronavirus disease 2019 (COVID-19) in a patient co-infected by HIV with a normal CD4^+^ T cell count

**DOI:** 10.1186/s12981-020-00301-3

**Published:** 2020-07-23

**Authors:** Wu Menghua, Zheng Xin, Liu Jianwei, Zhang Yu, Yao Qinwei

**Affiliations:** 1 Department of Urology, Beijing You’an Hospital, Capital Medical University, Beijing, China; 2 Department of Critical care medicine of liver disease, Beijing You’an Hospital, Capital Medical University, Beijing, China

**Keywords:** COVID-19, SARS-CoV-2, People living with HIV, RT-PCR

## Abstract

**Background:**

The COVID-19 has been a severe pandemic all around the world. Nowadays the patient with co-infection of HIV and SARS-CoV-2 was rarely reported. Here we reported a special case with HIV and SARS-CoV-2 co-infection, which showed a prolonged viral shedding duration.

**Case presentation:**

The patient was infected with HIV 8 years ago through sexual transmission and had the normal CD4^+^T cell count. She was found SARS-CoV-2 positive using real-time Polymerase Chain Reaction (RT-PCR) during the epidemic. Most importantly, the patient had a prolonged viral shedding duration of SARS-CoV-2 about 28 days.

**Conclusion:**

The viral shedding duration may be prolonged in people living with HIV. The 14 days isolation strategy might not be long enough for them. The isolation or discharge of these patients needs further confirmation for preventing epidemics.

## Background

COVID-19 is a novel virus disease with over 7250,000 confirmed cases worldwide [1]. And the knowledge regarding epidemiology and clinical presentation has been evolving gradually in the past months since the initial identification. In the general population, the reported case fatality rate ranged from 1.2 to 11.9% in different countries [2, 3]. Xu et al. [4] described that 113 patients had persistently positive PCRs results for at least 21 days. And Lu et al. [5] also reported a maximum 20 days of prolonged viral clearance period.

Here we reported a case of HIV and SARS-CoV-2 co-infection who had a prolonged viral shedding duration about 28 days.

### Case report

A 49-years old female diagnosed with HIV infection 8 years ago under regular ART (anti-retroviral therapy) went to our clinic for fatigue (day 1 of illness). He got a fever (day 4) with a maximum temperature of 38 ℃, accompanied by pharyngeal pain. The patient showed chills on day 5. Considering the clinical symptoms, the sputum sample was collected for RT-PCR assay of SARS-CoV-2 and the chest computed tomography (CT) was performed.

**Fig. 1:**
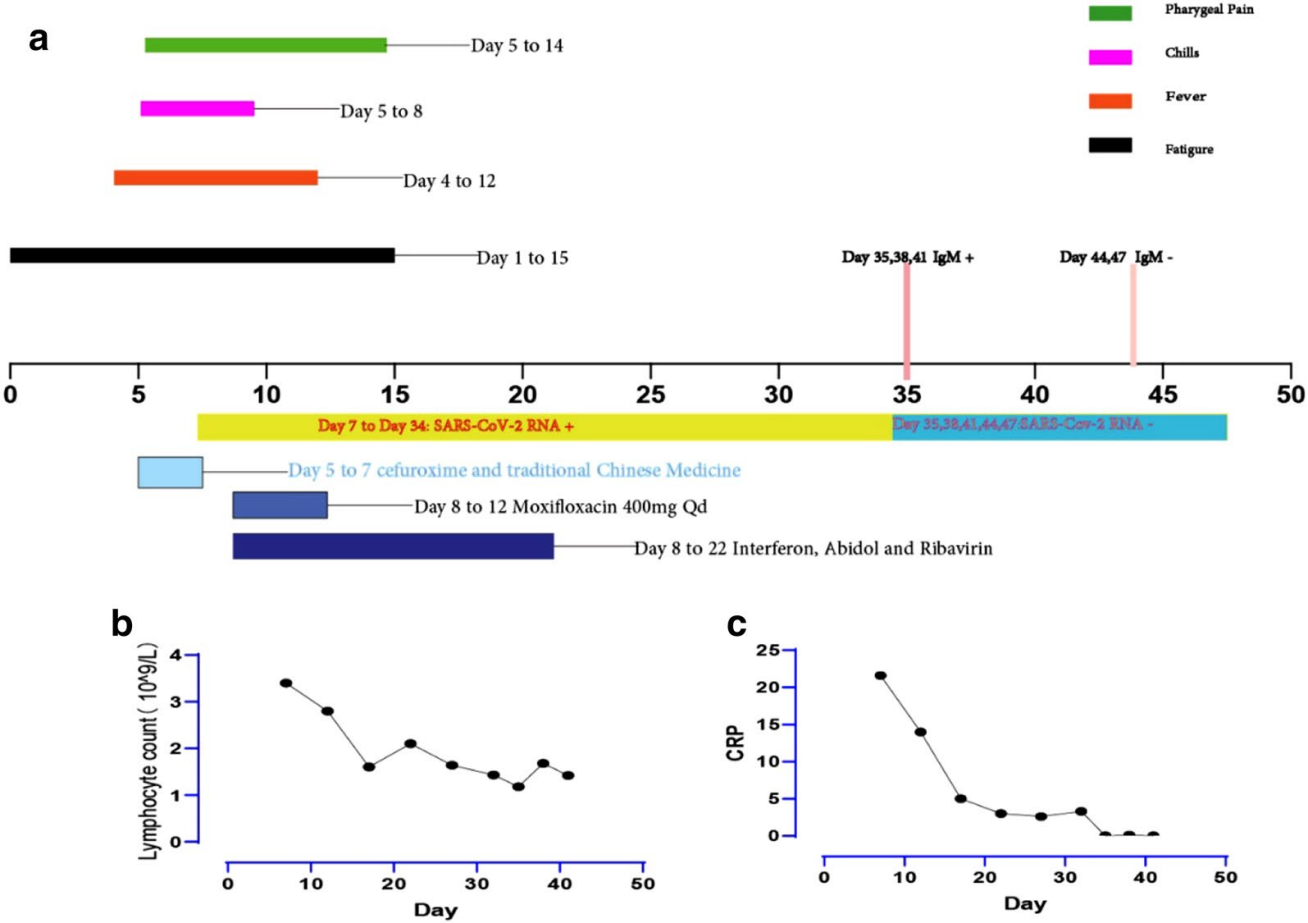
**a** The patient’s COVID-19 disease duration; **b** the Lymphocyte Count(10^9^/L); **c** C-reactive protein(mg/L)

**Fig. 2:**
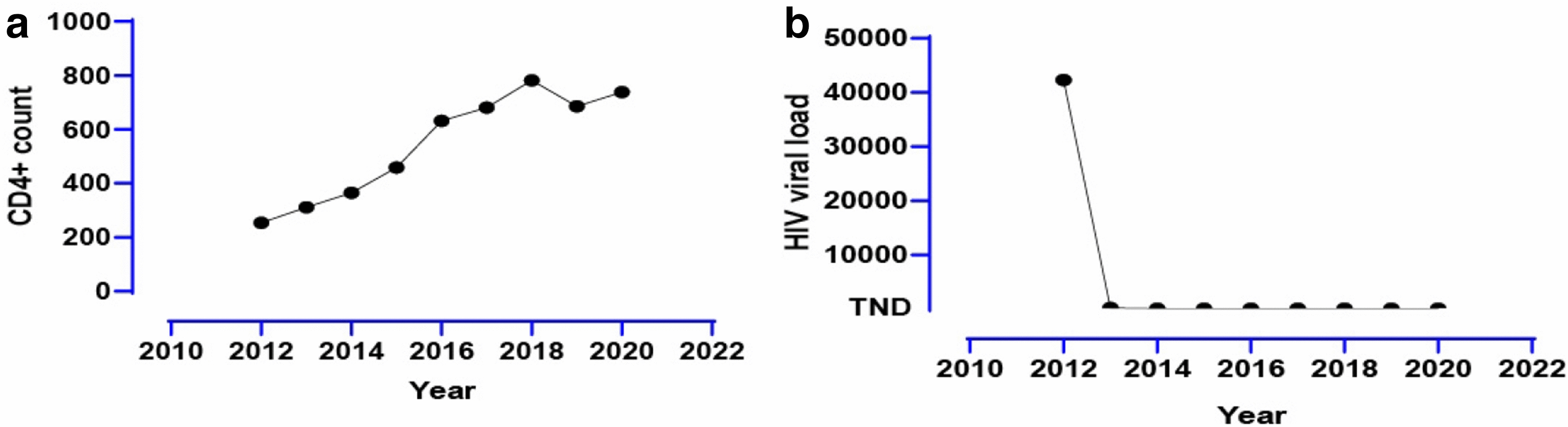
**a** The CD4^+^ T cell count; **b** the HIV viral load

Previous medical history included syphilis, which was cured. The ART is Efavirenz 600 mg, Zidovudine 300 mg, and Lamivudine 150 mg. After that, she continued the ART regularly. Although the nadir CD4 + T cell count was 224, a recent test was normal. The HIV viral load remained undetectable from 2013 (Figs. 1, 2).

**Fig. 3:**
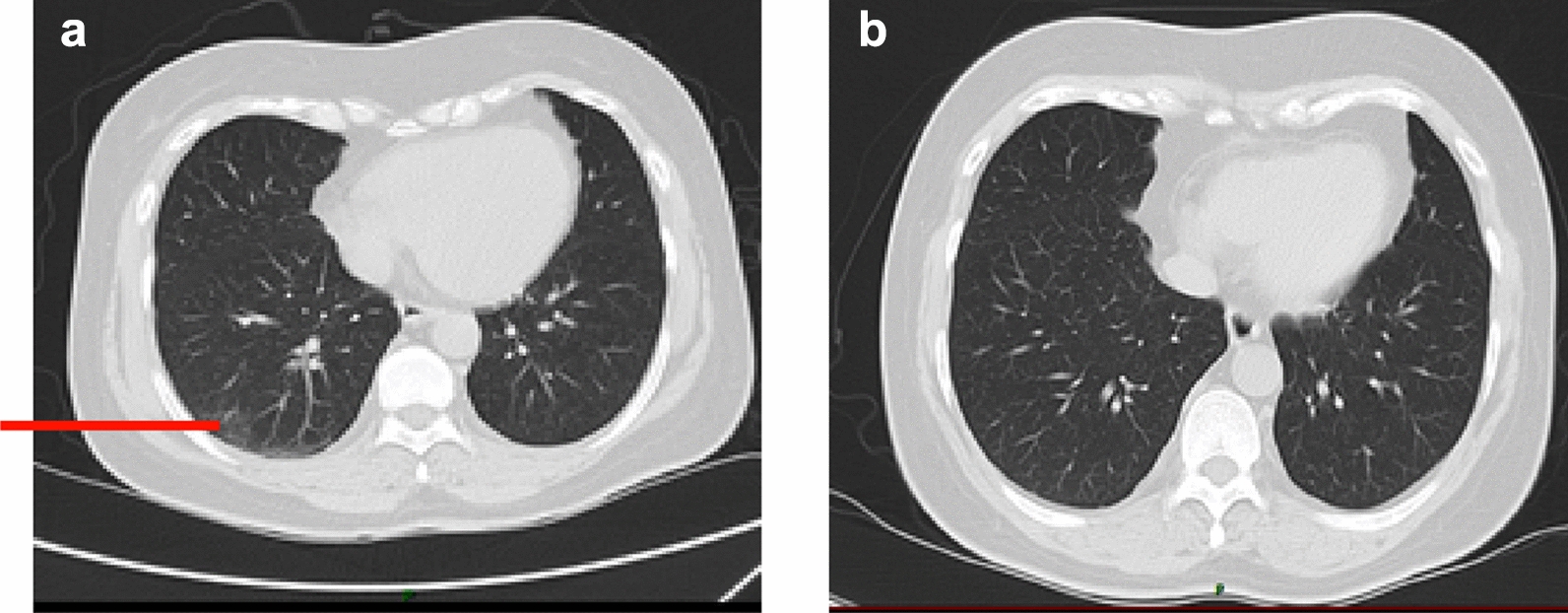
The CT scan on Day 5(**a**) and Day 15(**b**)

The CT result showed ground glass dense shadow and cord shadow under the pleura of the lateral segment of the middle lobe and dorsal-base segment of the lower lobe of the right lung (Fig. 3). Meantime, he was treated with cefuroxime and traditional Chinese medicine (Lianqin oral solution and Lianhua Qingwen capsule). At that time, the result of RT-PCR for SARS-CoV-2 was negative. But the symptoms were not relieved. We considered the possibility of false-negative to the RT-PCR result [6]. So, we had a re-check of RT-PCR for SARS-CoV-2 on day 7. The test result on day 7 turned positive, and the patient was diagnosed with COVID-19 (moderate type).

According to the Chinese COVID-19 treatment guideline at that time [7], on Day 8, we changed the cefuroxime and traditional Chinese medicine to interferon atomization (5 million bid), ribavirin (150 mg TID), and abidol (200 mg TID) for antiviral treatment. Meanwhile, the moxifloxacin (400 mg QD) was given to the patient for preventing bacterial infection.

On day 12, the temperature of the patient returned to normal. The symptoms of the patient alleviated completely, and the result of the CT scan on day 15 was also back to normal (Fig. 2). We consistently tested the RT-PCR for COVID-19 on day 19, day 25, and day 31 to 34, but all the results remained positive. The RT-PCR for COVID-19 turned negative for the first time on the day 35. Meantime, the IgM antibody for SARS-CoV-2 on day 35 was positive. Then we tested the RT-PCR and IgM for SARS-CoV-2 every 3 days. The RT-PCR for SARS-CoV-2 remained negative, while the IgM antibody for SARS-CoV-2 turned negative on day 44. We confirmed the viral clearance, and the patient was discharged on day 47.

## Discussion

Nowadays, the COVID-19 has been a worldwide epidemic disease. As an epidemic disease, viral shedding duration is the key to disease control. Some studies found asymptomatic people who were still carrying the virus after isolation for 14 days [8].

The prolonged viral shedding duration in the general population has been reported by several studies and the prolonged viral shedding duration reported ranges from 21 to 45 days [4, 9].

But there are few studies about the viral shedding duration in suspicious immunocompromised patients. At present, there are several cases of patients in immune suppressive status after organ transplantation. Huang reported two patients with COVID-19 who had undergone transplantation, one of whom had bone marrow transplantation, and the other had kidney transplantation. These two patients finally transferred to ARDS (Acute respiratory distress syndrome), and eventually died after [10]. Among the immunocompromised patients, Zhang et al. [11] reported a patient with kidney transplantation who had a prolonged viral shedding duration for 63 days.

This is the first report of a patient co-infected with HIV and SARS-CoV-2 who showed a prolonged viral shedding duration. Even though not all people living with HIV are immunocompromised, especially those under ART with an undetectable HIV viral and normal CD4 count, these people may still be vulnerable to viral infection or subsequent bacterial pneumonia than the general population. Further studies and data collection are needed for this.

In our case, the patient had a history of fever and had CT findings of viral pneumonia. COVID-19 was diagnosed by the positive result of RT-PCR. And the viral shedding duration lasted about 28 days.

Xu et al. [4] concluded the risk factors of prolonged viral RNA shedding in COVID-19 patients: male sex, delayed admission to hospital after illness onset, and invasive mechanical ventilation. These risk factors cannot explain the prolonged duration of the patient in our report.

Qin et al. [12] reported that the total number of B cells, T cells, and NK cells decreased significantly in patients with COVID-19. And the sum of lymphocytes of the severe group dropped more significantly than the moderate group. So, the infection of SARS-CoV-2 might be dramatically for the people living with HIV. On the one hand, the immune system might be impaired after the infection of SARS-CoV-2 by the depletion of T lymphocytes [12]. On the other hand, during the chronic phase of HIV infection, generalized immune activation, and systemic CD4^+^ T lymphocyte depletion occur. Fortunately, the CD4^+^ T cell count of our case was normal from 2016 to now, which might be the reason for not causing severe pneumonia. But the prolonged viral shedding duration.

The paradox between the prolonged viral shedding duration and moderate clinical course might be due to the impaired cellular function despite normal CD4 + T cell count in people living with HIV [13].

Lu et al. [5] revealed that prolonged viral RNA shedding in children was associated with symptomatic infection, fever, pneumonia, and lymphocyte count less than 2.0 × 10^9^/L. The lymphocyte count of our case was also less than 2.0 × 10^9^/L, which might be a co-factor for the prolonged viral shedding duration.

At last, there are no specific antiviral drugs for SARS-CoV-2. This patient was treated with ribavirin and abidol, which may not inhibit the replication of SARS-CoV-2 effectively.

## Conclusion

The viral shedding duration may be prolonged in people living with HIV. The 14 days isolation strategy might not be long enough for them. The isolation or discharge of these patients needs further confirmation for preventing epidemics.

## Data Availability

All data generated or analyzed during this study are included in this published article.
